# Treatment of Women

**Published:** 1881-07

**Authors:** 


### TREATMENT OF WOMEN.

From the fall of the Roman empire in the West to the fourteenth and fifteenth century, women spent most of their time alone, almost entirely strangers to the joys of social life; they seldom went abroad but to be spectators of such public diversions and amusements as the fashion of the times countenanced. Francis I. was the first who introduced women on public days to court; before his time nothing was to be seen in any of the courts in Europe but gray-bearded politicians, plotting the destruction of the rights and liberties of mankind, and warriors clad in complete armor, all ready to put their plots into execution. In the thirteenth and fourteenth centuries elegance had scarcely any existence, and even cleanliness was hardly considered as laudable. The use of linen was not known, and the most delicate of the fair sex wore woolen underclothing. In Paris they had meat only three times a week; and ten pounds was a large "portion" for a young lady. The better sort of citizens used splinters of wood and rags dipped in oil instead of candles, which in those days were a luxury rarely to be met with. Wine was only to be had at the shops of the apothecaries, where it was sold as a cordial; and to ride in a two-wheeled car was reckoned a grandeur so enviable that Philip the Fair prohibited the wives of citizens from enjoying it. In the time of Henry VIII. of England, the peers of the realm carried their wives behind them on horseback when they went to London, and in the same manner took them back to their country-seats, with hoods of waxed linen over their heads, and wrapped in mantles of cloth to secure them from the cold.

---

When the twenty-four hours of each day and night are numbered from one up to twenty-four, as proposed for the benefit of clockmakers, and to do away with a. m. and p. m. of the railroad time-tables, 21 o'clock will be considered as the shank of the evening, and 17 o'clock will be considered as the proper time for dinner.

---

### AFTER GRADUATION.

A few years ago a young man of promise was graduated at Harvard University. He determined to become a cotton manufacturer. Instead of relying upon his general education, and waiting for an opening, as many of his classmates did, he began at once to prepare specially for the business he had chosen, by entering a machine-shop as a workman, making full hours and acquainting himself with every part of the machinery of a cotton mill. From the machine-shop he went into the cotton-mill, and by hard work and close attention rapidly acquired a thorough knowledge of all the processes of cotton manufacture. While some of his classmates were waiting and looking for an opening in business, and others were with difficulty filling subordinate positions, he was rapidly rising, step by step, until he is, to-day, in charge of one of the largest cotton-mills in New England, with ample salary, and, what is better, is discharging the duties of his position with great satisfaction to the company he serves.—*Providence (R. I.) Journal.*

---

It is hard for an empty bag to stand upright.

There is no government so grievous as no government at all. Despotism may be terrible, but anarchy is diabolical.

